# Local Equilibrium in Transient Heat Conduction

**DOI:** 10.3390/e27020100

**Published:** 2025-01-21

**Authors:** Kirill Glavatskiy

**Affiliations:** School of Information and Physical Sciences, The University of Newcastle, Callaghan 2308, NSW, Australia; kirill.glavatskiy@newcastle.edu.au

**Keywords:** local equilibrium, irreversible thermodynamics, thermodynamic inertia

## Abstract

Extended irreversible thermodynamics (EIT) has been widely used to overcome the deficiencies of classical irreversible thermodynamics in describing fast transport phenomena. By employing fluxes as additional independent variables and rejecting local equilibrium hypothesis, EIT may provide a thermodynamically consistent framework for high-frequency and non-local processes. Here, we propose an alternative approach to EIT that shares the same objective but does not reject local equilibrium hypothesis. Using the rates of change of the energy density as the additional independent variable, we illustrate this approach for two typical problems of transient heat conduction: the Cattaneo-type flux model with thermodynamic inertia and the two-temperature model of energy transfer in a phonon–electron system.

## 1. Introduction

The classical formulation of irreversible thermodynamics (CIT) provides a consistent framework for the local description of a thermodynamic system in which quantities change with position and time [1]. It employs the hypothesis of *local equilibrium*, assuming that macroscopic thermodynamic relations (such as an equation of state or the Gibbs relation) are valid at the meso-scale. The validity of local equilibrium has been demonstrated in various homogeneous [1] and heterogeneous [2,3,4] systems and even at the nano-scale [5]. However, this question remains open for systems that involve high-frequency phenomena, such as the heating of solids via laser pulses, ultrasound in gases, phonon hydrodynamics, etc., in which local equilibrium appears to be violated [6]. Specifically, in such systems, the thermodynamic quantities at a certain position depend on the properties of the system at the adjacent positions, giving rise to various non-local descriptions [7]. Furthermore, the assumption of local equilibrium in such systems appears to violate the second law of thermodynamics. Such inconsistencies have been resolved by Extended irreversible thermodynamics (EIT), which provides a thermodynamically consistent framework for the description of fast transport phenomena at the micro-and nano-scale [6].

In this paper, we propose an alternative to EIT approach that does not reject local equilibrium hypothesis, while it still provides a thermodynamically consistent description. Following the steps of EIT, we demonstrate this approach for the famous Maxwell–Cattaneo–Vernotte (MCV) equation [8,9,10] and the two-temperature model of electron–phonon thermal coupling [7].

Let q(r,t) be the heat flux and T(r,t) the temperature in the system of interest, both of which depend on position and time. Using the dot over the symbol is used to indicate the partial time derivative, the MCV-type energy transport equation can be written as

(1) $$q_{C}+\tau {\dot{q}}_{C}=-\lambda \nabla T$$

where τ is the relaxation time, λ is the thermal conductivity, and the subscript C refers to the MCV-type behavior. Compared with Fourier’s law, $q_{F}=-\lambda \nabla T$, Equation (1) contains an additional term, $\tau \dot{q}$, which accounts for the so-called thermodynamic inertial effect. Such inertia is crucial in fast processes, where the transient dynamics demonstrate a delayed response.

Although Equation (1) accurately describes the dynamics of the aforementioned phenomena, it appears to violate the second law of thermodynamics. The latter states that the local entropy production σ, defined through the entropy balance equation

(2) $$\rho \;\dot{s}=-\nabla \cdot J_{s}+\sigma ,$$

and represented as the product of the heat flux q and the thermodynamic force X, must be non-negative. In CIT, $X_{CIT}=\nabla \left(1/T\right)$. If the heat flux is described by Fourier’s law, then $\sigma_{CIT}$ is non-negative, as expected:

(3) $$\sigma_{CIT,\;F}=q_{F}\cdot X_{CIT}=\mu_{CIT}\;q_{F}^{2}=\frac{1}{\mu_{CIT}}\;X_{CIT}^{2}$$

where $\mu_{CIT}\equiv 1/\left(\lambda T^{2}\right)$. However, if the heat flux is described by Equation (1), then the entropy production

(4) $$\sigma_{CIT,\;C}=\mu_{CIT}\;q_{C}^{2}-\tau \;q_{C}\cdot {\dot{q}}_{C}$$

may become negative, thus violating the second law of thermodynamics.

EIT proposes a modification to local thermodynamic equations by extending the number of independent variables, which control an irreversible evolution of a thermodynamic system [6]. In particular, in the case of heat conduction, EIT postulates that the entropy depends explicitly on the heat flux q, in addition to the classical dependence on the internal energy, *u*. This, in turn, modifies the Gibbs relation for the entropy, so for the specific (per unit of mass) quantities it reads as

(5) $$ds_{EIT}\left(u,q\right)=\frac{1}{\theta }\;du-\frac{\tau \mu_{EIT}}{\rho }\;q\cdot dq$$

where θ is the generalized temperature, which is the non-equilibrium analogue of the equilibrium temperature *T*, ρ is the material mass density, and $\mu_{EIT}\equiv 1/\left(\lambda \theta^{2}\right)$, where λ is the thermal conductivity. This results in the thermodynamic force having an additional term compared to CIT

(6) $$X_{EIT}=\nabla \frac{1}{\theta }-\tau \mu_{EIT}\;{\dot{q}}_{C}$$

and hence the entropy production restoring its non-negativity:

(7) $$\sigma_{EIT,\;C}=q_{C}\cdot X_{EIT}=\mu_{EIT}\;q_{C}^{2}=\frac{1}{\mu_{EIT}}\;X_{EIT}^{2}$$

The approach of EIT has been demonstrated to be consistent with various microscopic descriptions in non-equilibrium statistical physics, as well as specific applications of microscopic heat transfer. The dependence of the non-equilibrium entropy *s* on the flux q makes EIT a non-local theory.

In this paper, we propose an alternative approach, which provides a thermodynamically consistent description of fast phenomena that, however, is truly *local*. Our approach achieves the same objective as EIT, but keeps the local equilibrium hypothesis intact. We consider two typical problems that are relevant in this context: the MVC-type model (1) itself is derived in Section 2 and the so-called two-temperature model, which arises in the description of energy transfer in an electron–phonon system, is derived in Section 3.

## 2. Transient Heat Conduction

We assume that the non-equilibrium entropy (per unit of mass) depends on the internal energy (per unit of mass) and the time rate of change of the internal energy [11]:

(8) $$s\left(r,\;t\right)=s[u\left(r,\;t\right),\dot{u}\left(r,\;t\right)]$$

so that its change in some process is governed by the modified Gibbs relation

(9) $$ds\left(u,\dot{u}\right)=\beta \;du+\alpha \;d\dot{u}$$

where

(10) $$\begin{array}{cc} \beta (u,\dot{u})\equiv & {\left.\frac{\partial s}{\partial u}\right|}_{\dot{u}}\\ \alpha (u,\dot{u})\equiv & {\left.\frac{\partial s}{\partial \dot{u}}\right|}_{u} \end{array}$$

Given that β(u,0)=1/T, we may identify 1/β as the non-equilibrium temperature. The physical meaning of α will become apparent below. It is convenient, however, to write it as $\alpha \left(u,\dot{u}\right)=\tilde{\alpha }\left[\beta \left(u,\dot{u}\right),\dot{u}\right]$, so that

(11) $$d\alpha ={\left.\frac{\partial \tilde{\alpha }}{\partial \beta }\right|}_{\dot{u}}d\beta +{\left.\frac{\partial \tilde{\alpha }}{\partial \dot{u}}\right|}_{\beta }d\dot{u}$$

We next assume that ${\left(\partial \tilde{\alpha }/\partial \dot{u}\right)}_{\beta }=0$, so that the dependence of α on $\dot{u}$, if any, is only manifested through the corresponding dependence of the non-equilibrium temperature 1/β:

(12) $$\alpha \left(u,\dot{u}\right)=\tilde{\alpha }\left[\beta \left(u,\dot{u}\right)\right]$$

This assumption may be justified by viewing Equation (9) as the first-order approximation in the expansion of *s* in the powers of $\dot{u}$. Indeed, an explicit dependence of α on $\dot{u}$ would result in *s* being dependent on higher than linear terms in $\dot{u}$. Thus, Equation (12) implies linear dependence of the non-equilibrium entropy on the time rate of change of the internal energy.

Heat conduction is governed by the energy balance equation

(13) $$\rho \;\dot{u}=-\nabla \cdot q$$

Taking the time derivative of Equation (13), we obtain

(14) $$\rho \;\ddot{u}=-\nabla \cdot \dot{q}$$

Substituting Equations (13) and (14) in Equation (9), we obtain the time rate of change of the entropy density

(15) $$\rho \;\dot{s}=-\beta \;\nabla \cdot q-\alpha \;\nabla \cdot \dot{q}$$

which, after rearrangement, results in

(16) $$\rho \;\dot{s}=-\nabla \cdot \left(\beta q+\alpha \dot{q}\right)+\left(q+\dot{q}\;\frac{d\tilde{\alpha }}{d\beta }\right)\cdot \nabla \beta$$

Comparing it with the entropy balance Equation (2), we identify the entropy flux and the entropy production as

(17) $$\begin{array}{cc} J_{s}= & \beta \;q+\alpha \;\dot{q}\\ \sigma = & \left(q+\dot{q}\;\frac{d\tilde{\alpha }}{d\beta }\right)\cdot \nabla \beta \end{array}$$

In order for the entropy production to be non-negative, we must require

(18) $$q+\dot{q}\;\frac{d\tilde{\alpha }}{d\beta }=\frac{1}{\mu }\;\nabla \beta$$

where μ is positive. Comparing Equation (18) with MCV Equation (1), we note that these are the same equations. This essentially means that the assumptions (9) and (12) produce MCV Equation (1) in a thermodynamically consistent manner.

We can now identify the coefficients α and μ. Comparing again Equation (18) with (1), we identify $\mu =\beta^{2}/\lambda$, which thus has the same meaning as $\mu_{CIT}$ and $\mu_{EIT}$. Furthermore, we observe that $d\tilde{\alpha }/d\beta =\tau$, and hence

(19) $$\alpha (u,\dot{u})=\int \tau (\beta )\;d\beta$$

We will further refer to the combination

(20) $$\tilde{q}\equiv q+\dot{q}\;\frac{d\tilde{\alpha }}{d\beta }$$

as the *generalized flux*. Identifying the thermodynamic force as X≡∇β, we obtain for the entropy production

(21) $$\sigma =\tilde{q}\cdot X=\mu \;{\tilde{q}}^{2}=\frac{1}{\mu }\;X^{2}$$

## 3. Two-Temperature Model

We now consider a heterogeneous system that is made of two components with their own temperatures. A reference example of a such system is heat transport in metals, where one can observe the electron gas at the temperature $T_{e}$ and the phonon gas (the lattice) with the different temperature $T_{p}$ [7]. In such a system, heat is transferred in two modes: (i) via spatial heat fluxes $q_{e}$ and $q_{p}$ of electrons and phonons, respectively, and (ii) via a (scalar) heat flux $q_{ep}$ between the electron and phonon subsystems at the same position.

When the energy densities (per unit of volume) $u_{e}$ and $u_{p}$ of the corresponding subsystems are introduced, the energy balance equation for each of the subsystems is

(22) $$\begin{array}{cc} {\dot{u}}_{e}= & -\nabla \cdot q_{e}-q_{ep}\\ {\dot{u}}_{p}= & -\nabla \cdot q_{p}+q_{ep} \end{array}$$

We note that the thermodynamic behavior of the electron and phonon subsystems is different, resulting in certain simplifications for Equation (22). We will, however, carry the initial derivation in general terms (keeping the notation of the *e* and *p* subsystems), reducing to the particular cases later.

We next generalize Equation (8) so that the entropy density (per unit of volume) *s* of the system can be represented as a sum of the corresponding densities, $s_{e}$ and $s_{p}$ of the subsystems, each depending on the corresponding volumetric internal energy density and its time rate of change:

(23) $$s\left(r,\;t\right)=s_{e}\left[u_{e}\left(r,\;t\right),{\dot{u}}_{e}\left(r,\;t\right)\right]+s_{p}\left[u_{p}\left(r,\;t\right),{\dot{u}}_{p}\left(r,\;t\right)\right]$$

The corresponding Gibbs equation for the overall system takes the following form:

(24) $$ds\left(u_{e},u_{p},{\dot{u}}_{e},{\dot{u}}_{p}\right)=\beta_{e}\;du_{e}+\beta_{p}\;du_{p}+\alpha_{e}\;d{\dot{u}}_{e}+\alpha_{p}\;d{\dot{u}}_{p}$$

with β and α defined similarly to Equation (10), and α approximated similarly to Equation (12):

(25) $$\begin{array}{cc} \alpha_{e}\left(u_{e},{\dot{u}}_{e}\right)= & {\tilde{\alpha }}_{e}\left[\beta \left(u_{e},{\dot{u}}_{e}\right)\right]\\ \alpha_{p}\left(u_{p},{\dot{u}}_{p}\right)= & {\tilde{\alpha }}_{p}\left[\beta \left(u_{p},{\dot{u}}_{p}\right)\right] \end{array}$$

Following the same process as in Section 2, we obtain, for the balance of the entropy density, (2), identifying the entropy flux $J_{s}$ and the entropy production σ as

(26) $$\begin{array}{cc} J_{s}\equiv & \beta_{e}\;q_{e}+\beta_{p}\;q_{p}+\alpha_{e}\;{\dot{q}}_{e}+\alpha_{p}\;{\dot{q}}_{p}\\ \sigma \equiv & {\tilde{q}}_{e}\cdot \nabla \beta_{e}+{\tilde{q}}_{p}\cdot \nabla \beta_{p}+q_{ep}\left(-\beta_{e}+\beta_{p}\right)+{\dot{q}}_{ep}\left(-{\tilde{\alpha }}_{e}\left(\beta_{e}\right)+{\tilde{\alpha }}_{p}\left(\beta_{p}\right)\right) \end{array}$$

where the generalized fluxes ${\tilde{q}}_{e}$ and ${\tilde{q}}_{p}$ are defined similarly to Equation (20).

To ensure that σ is non-negative, and taking into account that scalar and vectorial fluxes do not couple, we must require that

(27) $$\begin{array}{cc} \nabla \beta_{e}= & \mu_{ee}\;{\tilde{q}}_{e}+\mu_{ep}\;{\tilde{q}}_{p}\\ \nabla \beta_{p}= & \mu_{pe}\;{\tilde{q}}_{p}+\mu_{pp}\;{\tilde{q}}_{p} \end{array}$$

and

(28) $$\begin{array}{cc} q_{ep}= & g_{00}\;\left(-\beta_{e}+\beta_{p}\right)+g_{01}\;\left(-{\tilde{\alpha }}_{e}\left(\beta_{e}\right)+{\tilde{\alpha }}_{p}\left(\beta_{p}\right)\right)\\ {\dot{q}}_{ep}= & g_{10}\;\left(-\beta_{e}+\beta_{p}\right)+g_{11}\;\left(-{\tilde{\alpha }}_{e}\left(\beta_{e}\right)+{\tilde{\alpha }}_{p}\left(\beta_{p}\right)\right) \end{array}$$

with the coefficients $\mu_{ee}$, $\mu_{ep}$, $\mu_{pe}$, $\mu_{pp}$ and $g_{00}$, $g_{01}$, $g_{10}$, $g_{11}$ being non-negative. Equations (27) and (28) represent heat transport equations for an isotropic two-temperature system. The identification of the transport coefficients is performed for each particular phenomenon below.

### 3.1. Electron–Phonon Coupling

The two-temperature model for the electron–phonon energy transfer is [7]

(29) $$\begin{array}{cc} C_{e}\;{\dot{T}}_{e}= & \nabla \cdot q_{e}-G\;\left(T_{e}-T_{p}\right)\\ C_{p}\;{\dot{T}}_{p}= & G\;(T_{e}-T_{p})\\ q_{e}+\tau_{F}\;{\dot{q}}_{e}= & -\lambda_{e}\;\nabla T_{e} \end{array}$$

Here, $C_{e}$ and $C_{p}$ are the volumetric heat capacities of the electrons and the phonons, respectively, $\lambda_{e}$ is the thermal conductivity of the electron gas, and *G* is the phonon–electron coupling factor describing the rate of energy transfer between electrons and phonons [12], while $\tau_{F}$ is the relaxation time of the electron gas calculated at the Fermi surface [13].

The first two of Equation (29) are the energy balance equations for the electron and phonon subsystems, respectively, i.e., the particular manifestations of Equation (22). We note that the phonon balance equation misses the $\nabla \cdot q_{p}$ term, which is due to the fact that spatial equilibrium in the phonon gas is reached much faster than the one in the electron gas. For the same reason, the transport equation for the phonon subsystem is missing, while the transport equation for the electron subsystem (the third equation in Equation (29)) has the form of MCV Equation (1). Comparing Equations (27) and (28) with Equation (29) allows us to identify the new quantities, similarly to how it has been done in Section 2.

We first identify $\beta_{e}=1/T_{e}$ and $\beta_{p}=1/T_{p}$ as the non-equilibrium temperatures of the electron and phonon gas. Furthermore, $\left(d{\tilde{\alpha }}_{e}/d\beta_{e}\right)\equiv \tau_{F}$, and $\alpha_{e}=\int \tau_{F}\left(\beta_{e}\right)d\beta_{e}$.

Similarly, $\alpha_{p}=\int \tau_{p}\left(\beta_{p}\right)d\beta_{p}$. We note, however, that since Equation (29) does not contain the transport equation for the phonon flux, $\tau_{p}$ is undefined. Because the phonon gas reaches equilibrium much faster than the electron gas, one may consider the thermal conductivity of the phonon gas $\lambda_{p}=\infty$ to be infinite (or, equivalently, the phonon thermal resistivity to be zero, $\mu_{pp}=0$). This, in turn, means that $\tau_{p}$ may be considered zero; hence, $\alpha_{p}=0$, and therefore, the corresponding term $\alpha_{p}\;{\dot{u}}_{p}$ is absent from the Gibbs relation (24).

Next, we observe that the spatial coupling coefficients $\mu_{ep}=\mu_{pe}=0$. Thus, Equation (27) reduces to a simple equation, $\nabla \beta_{e}=\mu_{ee}\;{\tilde{q}}_{e}$, which is equivalent to the third of Equation (29) with $\mu_{ee}\equiv 1/\left(\lambda_{e}T_{e}^{2}\right)$.

Finally, we observe that the electron–phonon coupling manifests as a single term, $q_{ep}=G\;\left(T_{e}-T_{p}\right)$, so that $g_{01}=g_{10}=g_{11}=0$ and $g_{00}\equiv G\;T_{e}\;T_{p}$.

### 3.2. Coupling Between Similar Components

Consider now the two-temperature model for a system that consists of “similar” components. We define such “similarity” as $\alpha_{e}$ and $\alpha_{p}$ having the same functional form; i.e., for any β

(30) $$\alpha_{e}\left(\beta \right)=\alpha_{p}\left(\beta \right)=\alpha \left(\beta \right)$$

In this case, assuming that $\beta_{e}$ and $\beta_{p}$ are not too different, we can further approximate

(31) $$\tilde{\alpha }\left(\beta_{e}\right)\approx \tilde{\alpha }\left(\beta_{p}\right)+\frac{d\tilde{\alpha }}{d\beta }\;\left(\beta_{e}-\beta_{p}\right)$$

Then, the scalar part of the entropy production in Equation (26) becomes

(32) $$\sigma^{\left(s\right)}=\left(-\beta_{e}+\beta_{p}\right)\;\left(q_{ep}+\frac{d\tilde{\alpha }}{d\beta }\;{\dot{q}}_{ep}\right)$$

where we have taken into account that, due to Equation (30), ${\tilde{\alpha }}_{e}\left(\beta_{p}\right)-{\tilde{\alpha }}_{p}\left(\beta_{p}\right)=0$. Therefore, Equation (28) simplifies to

(33) $$q_{ep}+\frac{d\tilde{\alpha }}{d\beta }\;{\dot{q}}_{ep}=g\;\left(-\beta_{e}+\beta_{p}\right)$$

where *g* is non-negative. With the “equilibrium” notation, it has the form of MCV Equation (1) for the flux $q_{ep}$ betwen two subsystems:

(34) $$q_{ep}+\tau_{ep}\;{\dot{q}}_{ep}=G\;\left(T_{e}-T_{p}\right)$$

with $g\equiv GT_{e}T_{p}$.

## 4. Discussion

We have proposed an alternative approach to EIT, which describes transient heat conduction in a thermodynamically consistent manner.

When the expressions for the entropy production from CIT, EIT, and the proposed approach (Equation (3), Equation (7), and Equation (21), respectively) are compared, it is interesting to note that the entropy production is represented by the same functional bi-linear form of thermodynamic forces and fluxes in all three approaches. The difference, however, lies in the explicit identification of those forces and fluxes. In particular, the heat flux $q_{F}$ in CIT is the solution of Fourier’s law, and the heat flux $q_{C}$ in EIT is the solution of the MCV equation, while the heat flux $\tilde{q}$ in the transient approach is the generalized flux, defined by Equation (20). Similarly, the thermodynamic force in CIT is ∇(1/T), and the thermodynamic force in EIT is $\nabla \left(1/\theta \right)-\tau \mu {\dot{q}}_{C}$, while the thermodynamic force in the transient approach is ∇β. This suggests the universality of the force-flux bi-linearity of the entropy production, while the choice of the approach is dictated by the particular problem of interest.

The proposed transient approach and EIT address the same type of phenomena while providing different solutions. The main difference between the two approaches is the utilization of local equilibrium. EIT is a non-local theory, with the local thermodynamic properties being dependent on the heat flux. In contrast, the proposed transient approach is a local theory, with the local thermodynamic properties being dependent on the time rate of change of the internal energy.

It may appear, however, that such a difference between the proposed transient approach and EIT is merely philosophical. Indeed, the additional independent variable, the heat flux, is measured locally, at the same position as the thermodynamic densities. While this may be true empirically, there exists an important conceptual difference. Thermodynamic density is evaluated at a point that is the center of a small volume element. In contrast, flux is evaluated at a point that is the boundary between two adjacent volume elements. Thus, employing the heat flux as an independent variable means involving two neighboring volume elements, while employing the time rate of change of the internal energy as an independent variable means involving a single volume element. The latter description is more succinct and thus may, in certain circumstances, be preferred.

Another important difference between EIT and the proposed transient approach is the behavior in the stationary state. There, the transient approach reduces to the classical irreversible thermodynamics. In contrast, EIT remains distinct from CIT with the stationary heat flux still being used as the independent thermodynamic variable. This is, in principle, not forbidden; however, it does appear to deviate from the original motivation of EIT to describe fast transient processes. This suggests that EIT may be better suited for low-frequency phenomena in spatially inhomogeneous systems, such as nano-systems [14], while the proposed transient approach is better suited to high-frequency transient processes in homogeneous environments.

Finally, the analysis of the two-temperature model may suggest a simple solution to the problem of non-equilibrium temperature raised in EIT. In particular, in non-equilibrium, different degrees of freedom may have different temperatures. This creates difficulties in the identification of the unique non-equilibrium temperature, θ. The proposed transient approach does not require the existence of the unique non-equilibrium temperature, sustaining its consistency with multiple non-equilibrium temperatures defined in the system.

## Data Availability

This work does not use or produce any data.
